# Machine learning-based integration develops a mitophagy-related lncRNA signature for predicting the progression of prostate cancer: a bioinformatic analysis

**DOI:** 10.1007/s12672-024-01189-5

**Published:** 2024-07-29

**Authors:** Caixia Dai, Xiangju Zeng, Xiuhong Zhang, Ziqi Liu, Shunhua Cheng

**Affiliations:** 1 Department of Urology, The Second Xiangya Hospital of Central South University, Changsha, 410011 Hunan China; 2 Department of Outpatient, The Second Xiangya Hospital of Central South University, Changsha, 410011 Hunan China; 3 Department of Acupuncture and Moxibustion, The First Hospital of Hunan University of Chinese Medicine, Changsha, Hunan China

**Keywords:** Prostate cancer, Bioinformatic analysis, lncRNA, Machine learning, Progression

## Abstract

Prostate cancer remains a complex and challenging disease, necessitating innovative approaches for prognosis and therapeutic guidance. This study integrates machine learning techniques to develop a novel mitophagy-related long non-coding RNA (lncRNA) signature for predicting the progression of prostate cancer. Leveraging the TCGA-PRAD dataset, we identify a set of four key lncRNAs and formulate a riskscore, revealing its potential as a prognostic indicator. Subsequent analyses unravel the intricate connections between riskscore, immune cell infiltration, mutational landscapes, and treatment outcomes. Notably, the pan-cancer exploration of YEATS2-AS1 highlights its pervasive impact, demonstrating elevated expression across various malignancies. Furthermore, drug sensitivity predictions based on riskscore guide personalized chemotherapy strategies, with drugs like Carmustine and Entinostat showing distinct suitability for high and low-risk group patients. Regression analysis exposes significant correlations between the mitophagy-related lncRNAs, riskscore, and key mitophagy-related genes. Molecular docking analyses reveal promising interactions between Cyclophosphamide and proteins encoded by these genes, suggesting potential therapeutic avenues. This comprehensive study not only introduces a robust prognostic tool but also provides valuable insights into the molecular intricacies and potential therapeutic interventions in prostate cancer, paving the way for more personalized and effective clinical approaches.

**Supplementary Information:**

The online version contains supplementary material available at 10.1007/s12672-024-01189-5.

## Introduction

Prostate cancer represents a pervasive and intricate challenge in the field of oncology, necessitating innovative approaches for effective prognosis, stratification, and treatment [1–4]. Over the recent years, the integration of machine learning techniques has emerged as a promising frontier in the quest to unravel the intricate dynamics of cancer, offering a nuanced comprehension of molecular intricacies and potential avenues for therapeutic interventions [5–8]. In this landscape, our research aims to pioneer a groundbreaking approach through machine learning-based integration, forging a mitophagy-related long non-coding RNA signature meticulously crafted to predict the progression of prostate cancer.

Mitophagy, an intricately regulated process selectively targeting damaged mitochondria for degradation, has garnered recognition for its pivotal role in maintaining cellular homeostasis and its implication in the progression of various cancers [9–11]. However, the interplay between mitophagy-related genes and long non-coding RNAs (lncRNAs) remains a relatively uncharted territory, especially within the context of prostate cancer. Seizing upon this research gap, our study endeavors to harness the formidable capabilities of machine learning. Our primary goal is to bridge this knowledge gap, leveraging the extensive dataset from The Cancer Genome Atlas Prostate Adenocarcinoma (TCGA-PRAD) to uncover a mitophagy-related lncRNA signature that carries profound prognostic significance.

The intricate dance between mitophagy-related genes and lncRNAs presents an intriguing puzzle yet to be fully deciphered in the prostate cancer landscape. Machine learning serves as a powerful ally in this quest, providing a sophisticated toolset to analyze the complexities embedded within vast datasets [12–14]. By integrating computational prowess with biological insights, our study aims to shed light on the molecular intricacies governing the progression of prostate cancer. The TCGA-PRAD dataset, with its wealth of genomic information, becomes a treasure trove for unveiling patterns and relationships that may have eluded traditional analytical approaches [15–17].

The envisioned mitophagy-related lncRNA signature holds the promise of becoming a transformative prognostic tool. As we embark on this exploration, the integration of machine learning techniques not only amplifies our analytical capabilities but also positions us on the frontier of precision oncology. The dynamic interplay between mitophagy-related lncRNAs, their impact on cancer progression, and the potential therapeutic implications form the crux of our investigation. By navigating through the intricacies of this complex landscape, our research endeavors to contribute not only to the scientific understanding of prostate cancer but also to the development of clinically relevant tools that can guide more personalized and effective interventions. In essence, this study represents a concerted effort to harness the synergy of machine learning and biological insights, paving the way for a new era in prostate cancer research and clinical management.

In our pursuit, we initiated a meticulous identification process, leveraging Pearson correlation analysis to discern lncRNAs intricately linked with mitophagy-related gene expression. The outcome, a pool of 2,097 lncRNAs, paved the way for subsequent Cox regression analyses, unveiling 54 lncRNAs significantly associated with prostate cancer prognosis. These prognostic-related mitophagy-related lncRNAs (PRMR-LncRNAs) became the linchpin of our research, promising insights into the complex dynamics of prostate cancer progression. The integration of diverse machine learning algorithms became imperative for refining and validating our mitophagy-related lncRNA signature. A strategic selection process, considering 101 machine learning methods, culminated in the identification of 15 PRMR-LncRNAs using the StepCox[both] + RSF algorithm. This precision-driven approach not only optimized the predictive capabilities of our signature but also positioned it as a robust tool for discerning the intricate landscape of prostate cancer prognosis. As we delved deeper into the functional implications of our 54 PRMR-LncRNAs, Gene Set Variation Analysis (GSVA) illuminated their association with key biological pathways [18, 19]. The exploration of immune signaling pathways and the identification of the RIG-I like receptor signaling pathway as a significant correlate hinted at the broader role these lncRNAs play in shaping the tumor microenvironment [20, 21]. This functional insight not only enhanced our understanding of the molecular underpinnings but also hinted at potential therapeutic avenues.

Recognizing the inherent heterogeneity within prostate cancer, we turned to Non-negative Matrix Factorization (NMF) clustering [22–24]. Leveraging the expression patterns of our 54 PRMR-LncRNAs, we identified four distinct subtypes, each carrying unique prognostic implications. This nuanced subtyping not only presented an opportunity for more personalized prognosis but also hinted at the diverse molecular landscapes underlying prostate cancer progression. To ensure the clinical applicability of our findings, we constructed and validated a nomogram model based on the expression levels of the four key PRMR-LncRNAs. Calibration curves, Kaplan–Meier survival analyses, and time-dependent ROC curves collectively validated the robust predictive power of our signature. This step was crucial, bridging the translational gap from comprehensive molecular insights to actionable clinical tools. Weighted Gene Co-expression Network Analysis emerged as a pivotal tool to dissect the interplay between riskscore and various biological functions [25–27]. Immune cell infiltration analysis, employing multiple algorithms, further illuminated the intricate relationship between riskscore, Th1 cells, and the identified PRMR-LncRNAs [28–31]. This dual approach not only strengthened the reliability of our findings but also shed light on the potential immunomodulatory roles of the mitophagy-related lncRNA signature.

Our exploration extended beyond prognostic insights, delving into the realm of therapeutic tailoring. By predicting drug sensitivities based on riskscore, we identified drugs such as Carmustine and Entinostat with distinct suitability for high and low-risk group patients, respectively [32–37]. Molecular docking analyses further validated potential interactions between Cyclophosphamide and proteins encoded by mitophagy-related genes, suggesting novel therapeutic targets.

In conclusion, our research represents a comprehensive journey into the intricacies of prostate cancer progression. From the identification of mitophagy-related lncRNAs to the development of a robust signature, our approach combines machine learning precision, functional insights, and clinical validation. As we unravel the interplay between riskscore, immune modulation, and drug sensitivities, our findings not only deepen our understanding of prostate cancer but also pave the way for more targeted and personalized clinical interventions. This study serves as a beacon, guiding future research endeavors toward unraveling the complex tapestry of cancer progression.

## Methods

### Data acquisition and preprocessing

We downloaded the bulk transcriptome data and corresponding clinical data for PRAD from The Cancer Genome Atlas (TCGA, https://portal.gdc.cancer.gov/) database. All samples with incomplete information were excluded from the analysis. Sequencing data were converted into Transcripts per Million (TPM) format for subsequent analysis. For genes with multiple records, the mean value of these records was used. The TCGA database permits unrestricted access and analysis of its data by researchers. Our data processing workflow complies with TCGA guidelines and does not involve any ethical issues.

### Machine learning-driven development of a mitophagy-related lncRNA signature

In this investigation, machine learning techniques were applied to the TCGA-PRAD dataset to intricately develop a predictive signature amalgamating mitophagy-related lncRNAs, offering a profound avenue for evaluating the progression of prostate cancer [38, 39].

### Identification of PRMR-LncRNAs

Within the TCGA-PRAD cohort, the identification process involved the adept utilization of Pearson correlation analysis, revealing 2,097 lncRNAs intricately correlated with mitophagy-related gene expression. Subsequently, Cox regression analysis was performed on these lncRNAs using the "survival" package, incorporating parameters such as overall survival (OS) and progression-free survival (PFS). This analysis identified 54 mitophagy-related lncRNAs (prmr-lncRNA).

### NMF clustering for subtyping prostate cancer patients

A sophisticated application of NMF clustering, leveraging the expression patterns of the 54 PRMR-LncRNAs and patient survival data, led to the classification of prostate cancer patients into four distinct subtypes, each bearing significant differences in prognosis [22, 40]. We performed the above analysis using the "NMF" package.

### Integration of machine learning for LncRNA selection

The rigorous process of LncRNA selection involved the deployment of a repertoire of 101 machine learning algorithms, culminating in the identification of 15 PRMR-LncRNAs from the initial 54 candidates. StepCox[both] + RSF was selected based on its highest average consistency index (C-index) in the training and test datasets.

### Functional and pathway enrichment analysis of PRMR-LncRNAs

Exploration of the biological functions and pathways associated with the four candidate PRMR-LncRNAs was conducted using GSVA on the TCGA-PRAD samples [41, 42]. This insightful analysis, implemented with the “GSVA” package, extended to the investigation of their potential involvement in immune signaling pathways.

### Survival analysis in different subgroups

Stratification of TCGA-PRAD samples based on prostate-specific antigen (PSA) levels and initial treatment outcomes paved the way for a comprehensive survival analysis of the four key LncRNAs in diverse subgroups, unraveling their nuanced prognostic significance.

### Construction and validation of PRMR-LncRNAs signature

A nomogram model, constructed on the basis of the expression levels of the four key PRMR-LncRNAs through “rms” package, emerged as a robust tool for predicting the prognosis of prostate cancer. The validation of this model involved a meticulous process, encompassing calibration curves, Kaplan–Meier survival analysis, and time-dependent ROC curves. In ROC curves, the area under the curve (AUC) was greater than 0.6, which was considered to have good testing efficiency.

### Weighted gene co-expression network analysis (WGCNA)

The intricate correlation between riskscore and various biological functions was deciphered through the adept utilization of WGCNA, implemented with the “WGCNA” package [43, 44]. This comprehensive analysis unveiled potential associations between riskscore and functions such as RNA splicing, mRNA processing, and immune-related functions.

### Immune cell infiltration analysis

An extensive exploration of immune cell infiltration was conducted through various algorithms, including ssGSEA, XCELL, TIMER, QUANTISEQ, MCPCOUNTER, EPIC, and CIBERSORT [30, 45, 46]. This multifaceted analysis shed light on the intricate relationship between riskscore, Th1 cells, and the four PRMR-LncRNAs.

### Drug sensitivity prediction and molecular docking analysis

Utilizing the oncopredict package, drug sensitivity analysis based on riskscore revealed drugs such as Carmustine with distinct suitability for high-risk group patients and Entinostat for low-risk group patients. Additionally, molecular docking analyses predicted potential interactions between Cyclophosphamide and six mitophagy-related genes, paving the way for novel therapeutic targets.

### Statistical analysis

All analyses were conducted using R software (V4.1.3). The student t-test was deployed for normally distributed continuous variables, while the Mann–Whitney U-test was employed for non-normally distributed continuous variables. Differential expression analysis utilized the limma package (V3.58.1), with a predefined threshold of |logFC|> 0.5 and p < 0.05. The time-dependent ROC curve is plotted using the "timeROC" package. The KM curve is plotted using the "survival" package. A p-value less than 0.05 is considered statistically significant.

## Results

### Unraveling mitophagy-related lncRNA signatures in prostate cancer progression through machine learning integration

**Fig. 1:**
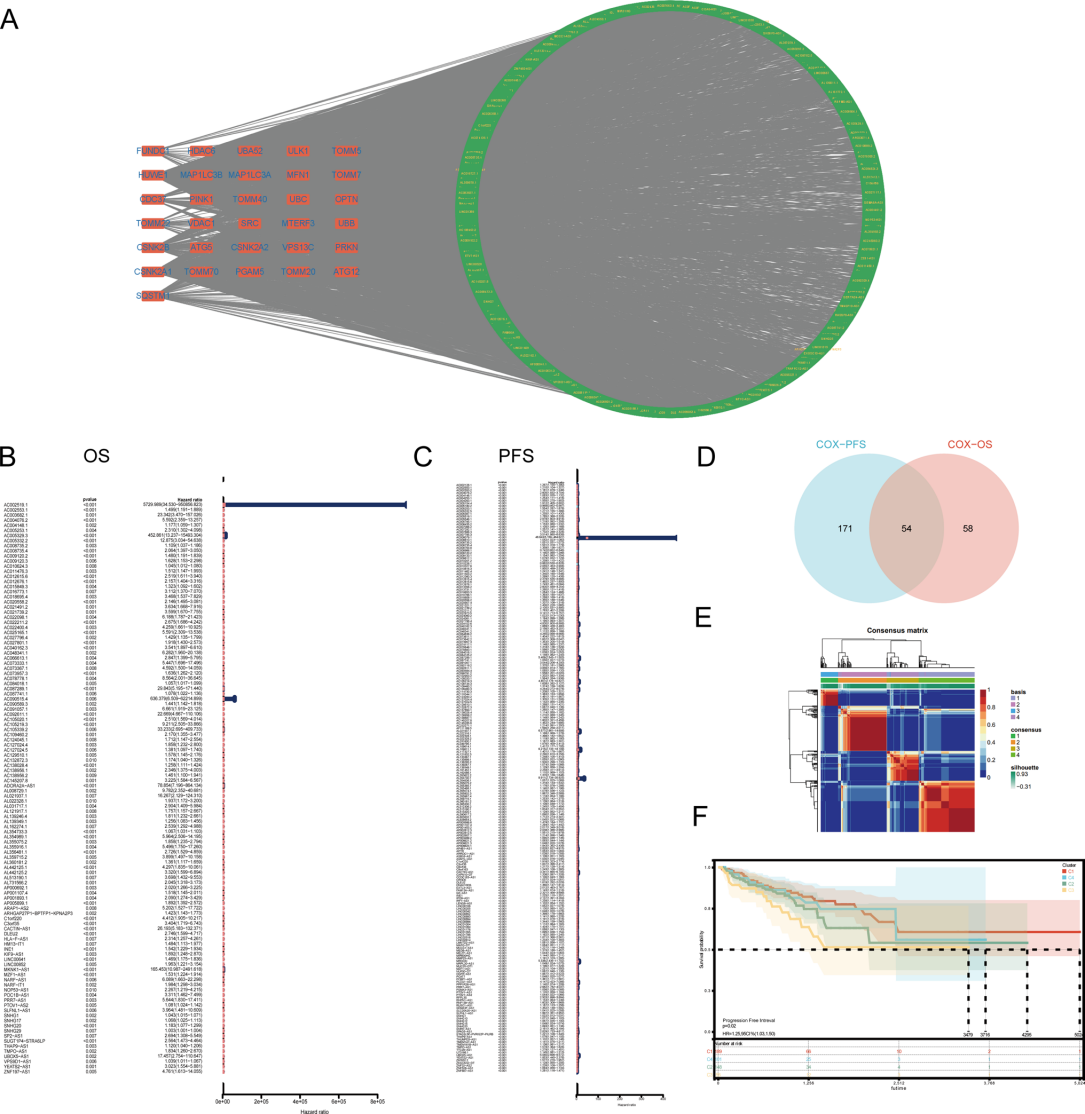
Identification and clustering of prognostic Mitophagy-related LncRNAs. **A** Network diagram of mitophagy-related genes and related LncRNAs. **B** Forest plot of Mitophagy-related LncRNAs with prognostic significance at p < 0.01 based on OS. **C** Forest plot of Mitophagy-related LncRNAs with prognostic significance at p < 0.01 based on PFS. **D** Venn diagram of the intersection between OS group and PFS group. **E** NMF clustering, k = 4. **F** K–M curves for PFS in different NMF clusters

In the confines of the TCGA-PRAD cohort, we deployed Pearson correlation analysis to meticulously identify 2,097 lncRNAs exhibiting a substantial correlation with the expression of mitophagy-related genes (correlation coefficient > 0.4, p < 0.05) (Fig. 1A). Subsequent univariate Cox regression analyses, incorporating OS and PFS as prognostic parameters, elucidated the prognostic significance of Mitophagy-related LncRNAs (MR-LncRNAs). Intriguingly, 112 and 225 MR-LncRNAs exhibited significant associations with OS and PFS, respectively (Fig. 1B, C). Through the intersection of these sets, we identified 54 MR-LncRNAs robustly correlated with prostate cancer prognosis, denoted as PRMR-LncRNAs (Fig. 1D). Subsequently, grounded in the expression profiles and survival data of these 54 PRMR-LncRNAs, NMF clustering on the TCGA-PRAD patients revealed four distinct subtypes with noteworthy differences (Fig. 1E).

Validation of our molecular subtyping conclusions ensued, employing progression-free interval (PFI) as a prognostic parameter and generating Kaplan–Meier survival curves for each subtype. Remarkably, substantial differences in prognostic trends among the four subtypes were observed (p = 0.02) (Fig. 1F). This thorough analysis underscores the pivotal role of the identified PRMR-LncRNAs in delineating distinct prognostic subtypes within the TCGA-PRAD cohort.

**Fig. 2:**
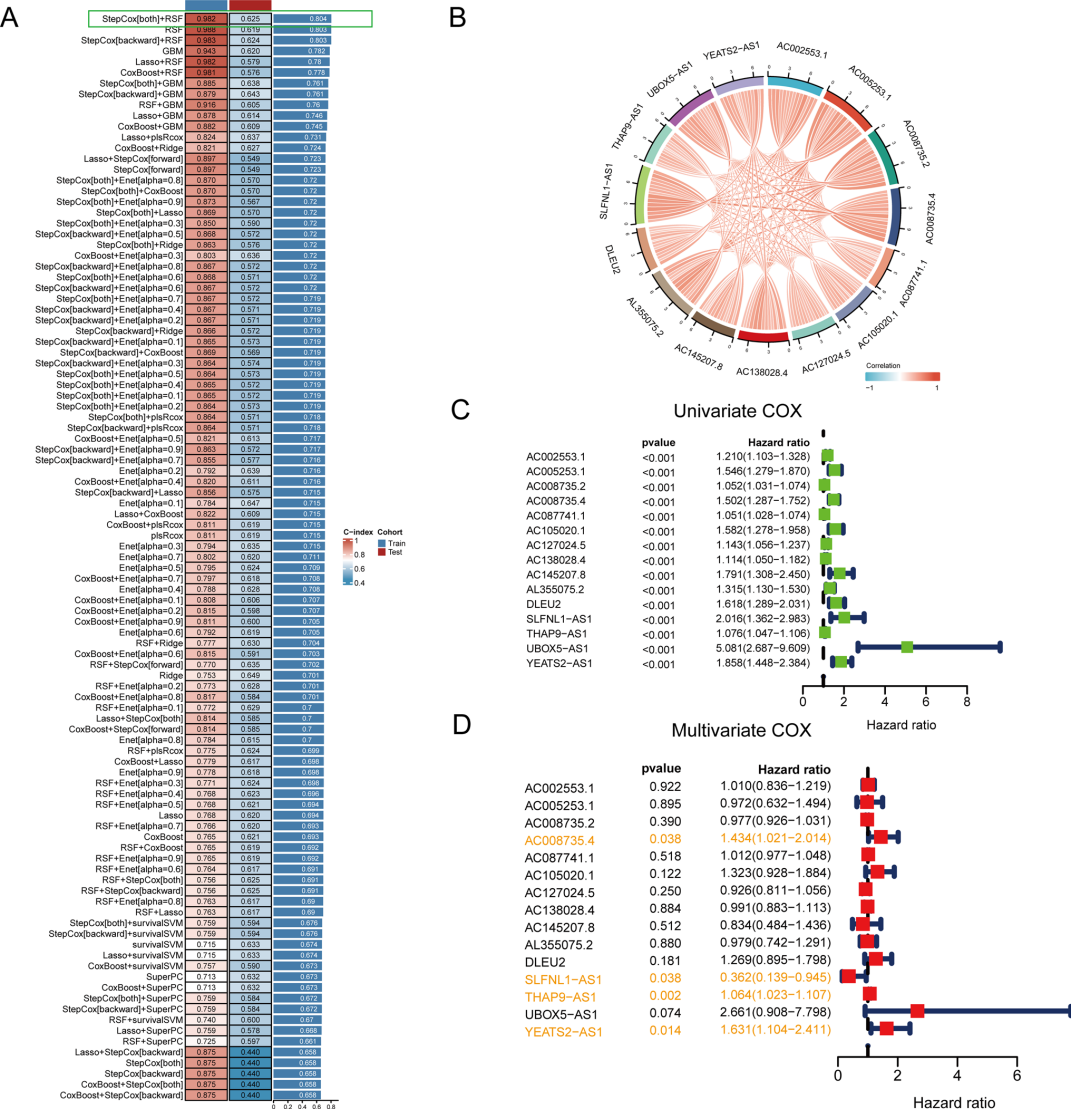
Filtrating candidate PRMR-LncRNAs for construction of PRMR-LncRNAs signature. **A** A total of 101 kinds of prediction models via LOOCV framework and further calculated the C-index of each model across all validation datasets. **B** Co-expression network within Mitophagy-related LncRNAs. **C** Univariate survival analysis of Mitophagy-related LncRNAs based on PFS event. **D** Multivariate survival analysis of Mitophagy-related LncRNAs based on PFS event

For the identification of pertinent lncRNAs for subsequent modeling, we devised an extensive strategy amalgamating diverse machine learning approaches. Initially, the TCGA-PRAD cohort underwent random division into training and testing sets. Subsequently, a comprehensive set of 101 machine learning methods was employed to discern lncRNAs most intricately linked with the prognosis of PRAD. Performance evaluation, gauged through the C-index, showcased that the StepCox[both] and Random Survival Forest (RSF) combination yielded the highest C-index, reaching 0.982 and 0.625 in the training and testing sets, respectively (Fig. 2A). Focus then shifted to the 15 MR-LncRNAs identified by StepCox[both] + RSF. Exploring their expression patterns through a correlation chord diagram, a remarkably intricate interplay among these lncRNAs emerged (Fig. 2B). PFS was selected as the prognostic parameter for subsequent analysis. Through single and multi-factor regression analyses on the 15 lncRNAs, four lncRNAs—AC008735.4, SLFNL1-AS1, THAP9-AS1, and YEATS2-AS1—surfaced as significant predictors with p-values less than 0.05 in both regression analyses (Fig. 2C, D). These four lncRNAs were subsequently designated as the final set for modeling purposes.

To garner deeper insights into the potential significance of the 15 selected lncRNAs, we conducted an exhaustive exploration of their expression patterns. Employing correlation chord diagrams, we visually depicted the intricate interconnections among these lncRNAs, offering a comprehensive understanding of their collaborative roles (Fig. 2B). This intricate network of interactions hints at a potential synergistic impact on mitophagy-related processes in prostate cancer progression. The identification of AC008735.4, SLFNL1-AS1, THAP9-AS1, and YEATS2-AS1 as pivotal lncRNAs with prognostic significance underscores their potential as robust predictors of prostate cancer progression. In the model, the expression of AC008735.4, SLFNL1-AS1, THAP9-AS1 and YEATS2-AS1 genes in different PCA cell lines showed significant differences (Figure S1). These lncRNAs, meticulously selected through the integration of machine learning techniques, are poised to form the foundation of a predictive model capable of enhancing our understanding of mitophagy-related mechanisms in the context of prostate cancer.

### Exploring the functional associations of the identified PRMR-LncRNAs in prostate cancer progression

**Fig. 3:**
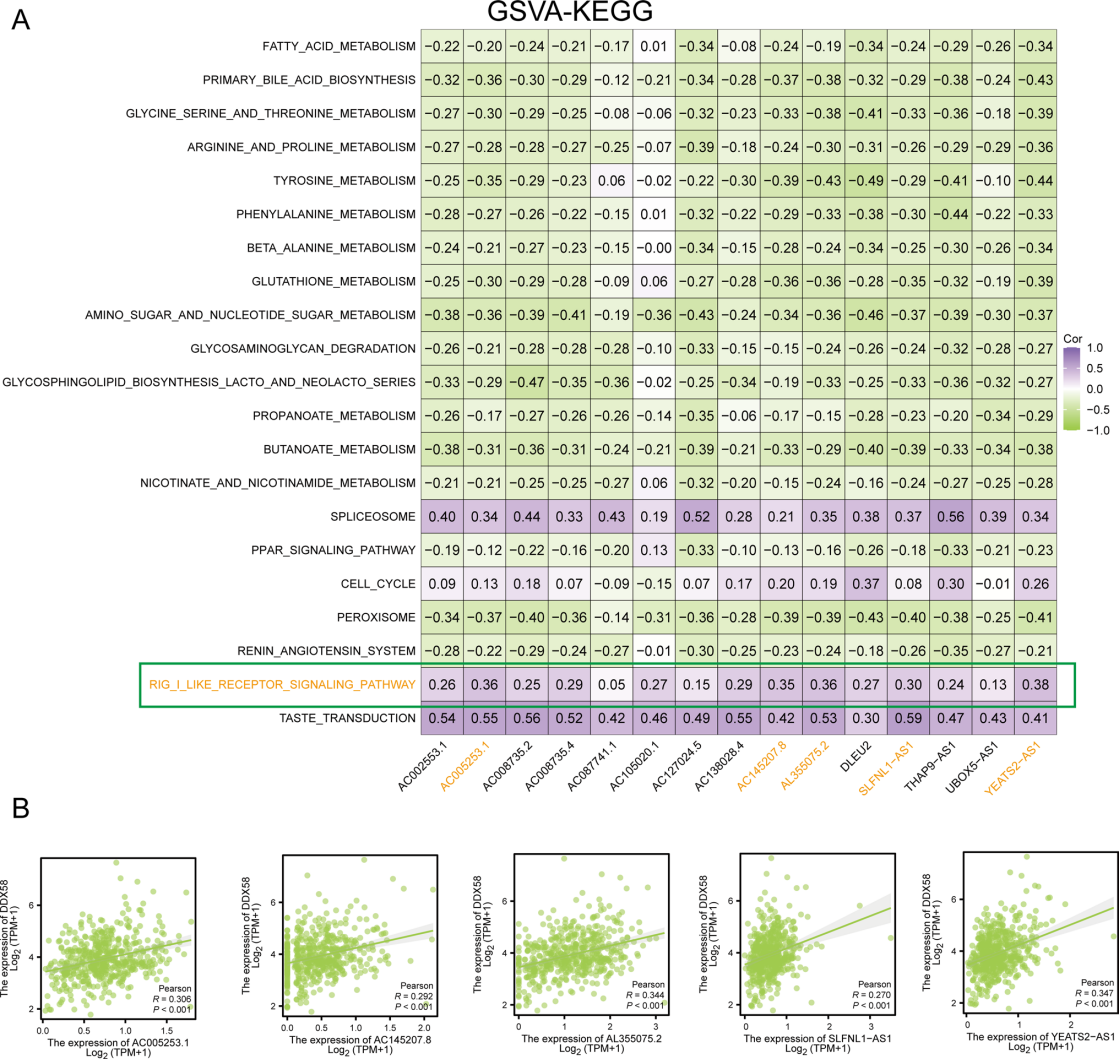
Exploration of underlying functions which candidate PRMR-LncRNAs are associated with. **A** Heat map of correlation between 15PRMR-LncRNAs and GSVA scores. **B** Scatter plot of association

To delve comprehensively into the functional implications of the four candidate PRMR-LncRNAs and comprehend their intricate contributions to the prognosis of PRAD, we executed GSVA utilizing the GSEA-C5 KEGG gene set on TCGA-PRAD samples. The expression levels of the 15 PRMR-LncRNAs, as identified by StepCox[both] + RSF, were subsequently subjected to Pearson correlation analysis with GSVA scores across various pathways. A heatmap visualizing the top 20 pathways, exhibiting the highest correlation, accentuated a particularly significant association with the RIG-I like receptor signaling pathway (Fig. 3A).

The RIG-I like receptor signaling pathway surfaced as the most prominently correlated with the expression of the 15 PRMR-LncRNAs. To glean deeper insights, our focus zeroed in on the core protein of this pathway, DDX58. A correlation scatter plot was subsequently generated, delineating the expression correlation between LncRNAs and DDX58 (Fig. 3B). This intricate analysis sheds light on the potential regulatory role of the identified PRMR-LncRNAs, particularly within the nuanced dynamics of the RIG-I like receptor signaling pathway.

In a concerted effort to gain profound insights into the prognostic significance of the four pivotal lncRNAs, survival analyses were conducted on samples from the TCGA-PRAD cohort, stratified into diverse subgroups based on PSA levels and the outcomes of initial treatments. This meticulous exploration sought to elucidate how these identified LncRNAs might manifest varying prognostic values across distinct clinical scenarios.

**Fig. 4:**
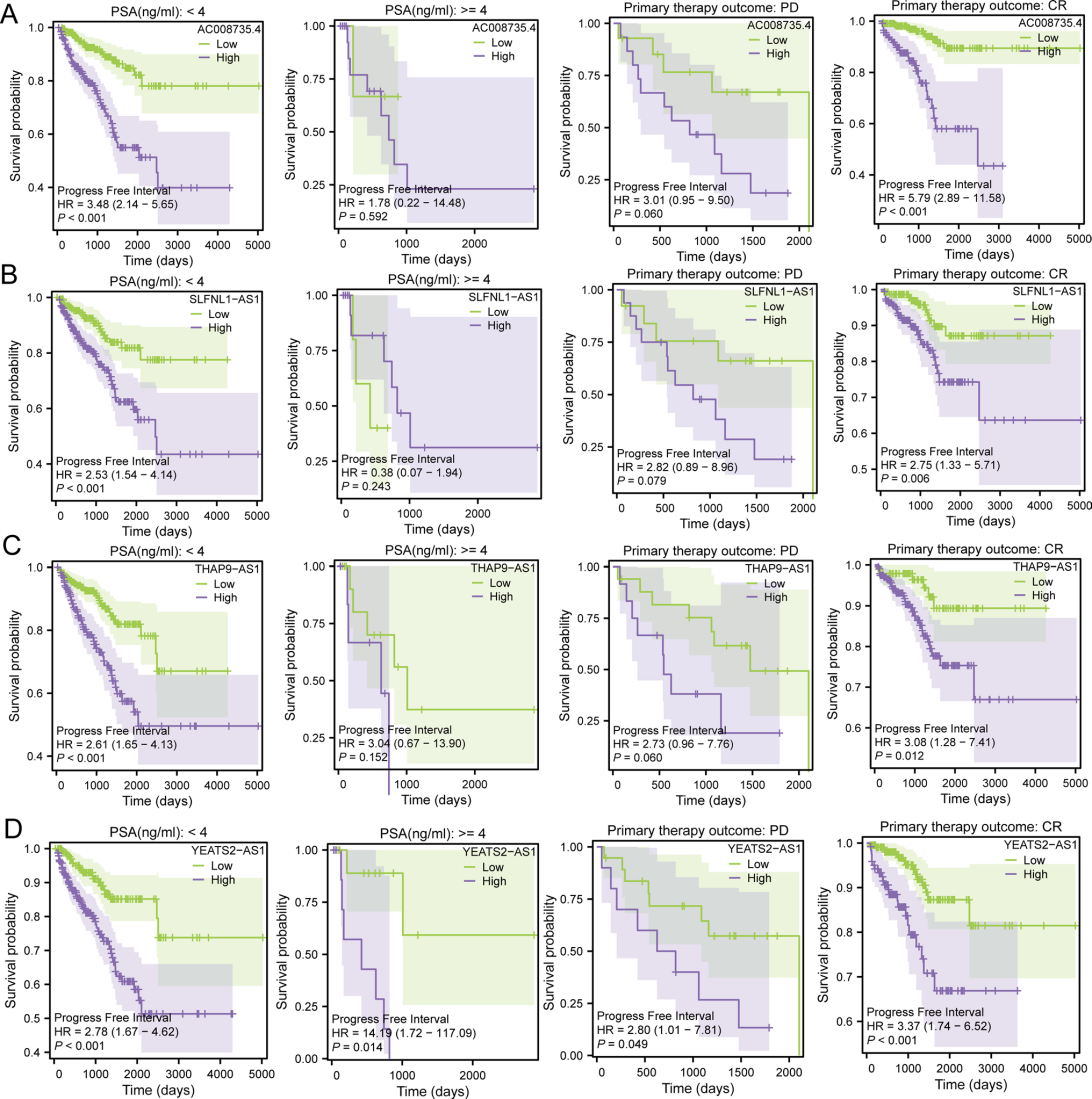
Survival analysis of the four key LncRNAs in different subgroups. **A** AC008735.4; **B** SLFNL1-AS1; **C** THAP9-AS1; **D** YEATS2-AS1

The analysis unveiled intriguing patterns in the prognostic value of the four key LncRNAs within specific subgroups. Remarkably, in patients exhibiting PSA levels below 4 ng/ml and those achieving a complete response (CR) as the outcome of initial treatment, the predictive efficacy of these LncRNAs was significantly enhanced. This suggests that the four key LncRNAs may wield superior prognostic efficacy in the realm of early-stage and less aggressive PRAD. The graphical representation of these findings is depicted in Fig. 4A–D.

The observed refinement in prognostic predictions for patients with PSA levels below 4 ng/ml and those with a CR to initial treatment underscores the potential of the four key LncRNAs in predicting favorable outcomes, particularly in cases of early-stage and less malignant prostate cancer. These findings carry profound implications for tailoring prognostic models that cater to specific clinical contexts, thereby advancing the precision and personalized nature of prostate cancer prognosis.

### Establishing a robust nomogram for prognostic prediction in prostate cancer

**Fig. 5:**
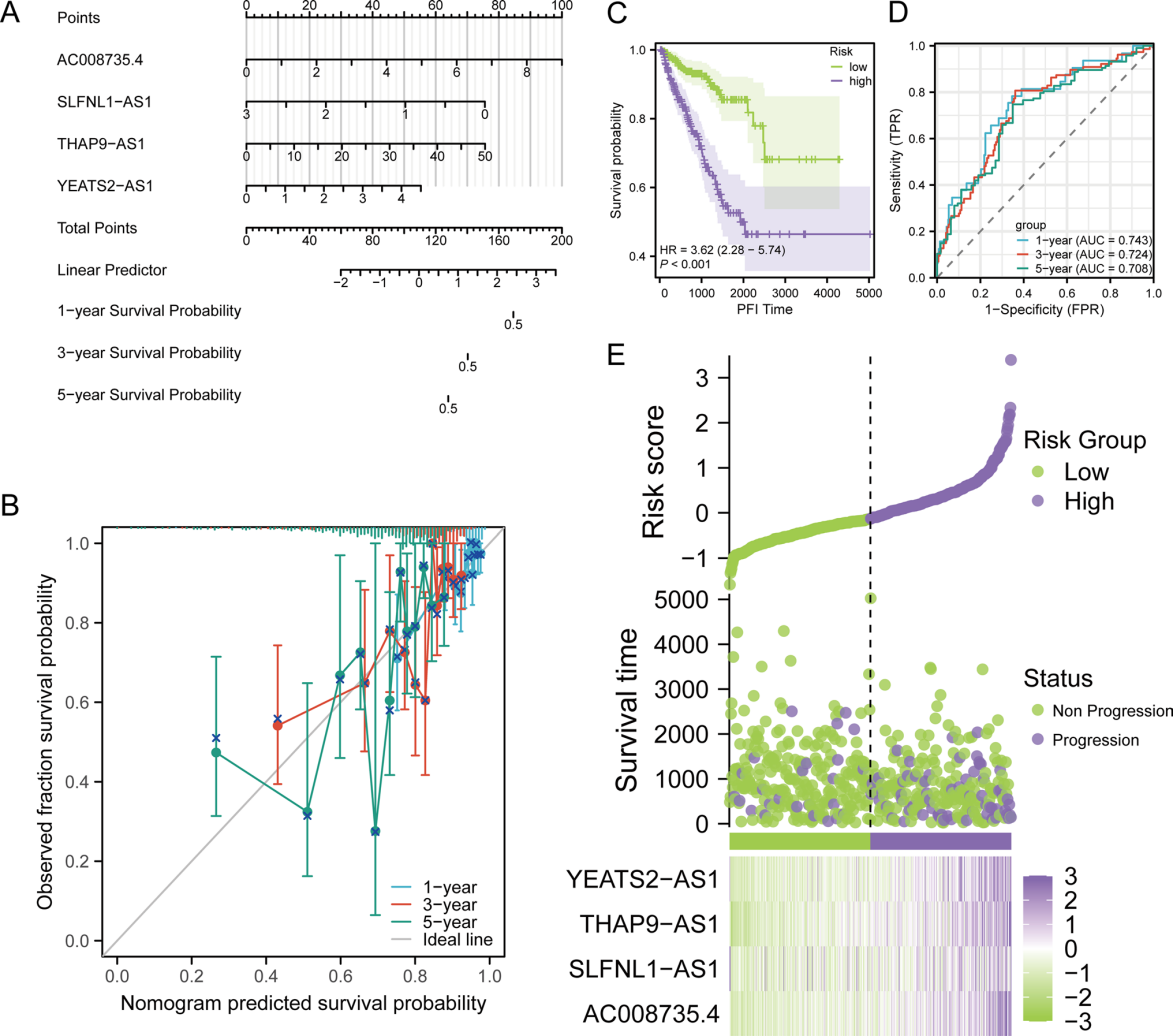
Construction and validation of PRMR-LncRNAs signature. **A** Nomogram prognostic model. **B** 1-year, 3-year, and 5-year calibration curves. **C** K–M curves for OS in high-risk and low-risk patient groups. **D** ROC analysis to evaluate the performance of signature in 1-year, 3-year, and 5-year prognoses. **E** Visualization of risk factors

Given the substantial research significance ascribed to the quartet of key lncRNAs delineated in our investigation, we initiated the development of a prognostic model tailored to prognosticate the trajectory of PRAD. Utilizing the expression profiles of AC008735.4, SLFNL1-AS1, THAP9-AS1, and YEATS2-AS1, we devised a nomogram designed to anticipate prognostic outcomes at 1, 3, and 5 years (Fig. 5A). To validate the nomogram's reliability, a risk score, contingent upon the expression levels of the four pivotal LncRNAs, was computed. Calibration of the nomogram ensued, and the resultant calibration curve (Fig. 5B) showcased minimal deviation from the standard line across the 1, 3, and 5-year intervals. This meticulous calibration substantiates the nomogram’s credibility in forecasting the progression of PRAD.

For an in-depth assessment of the prognostic prowess inherent in our nomogram, PRAD samples were categorized based on nomogram-derived risk scores. Subsequent stratification into high and low-risk groups facilitated the generation of Kaplan–Meier survival curves for the PFI parameter. The stark contrast in survival outcomes between the two groups (p < 0.001) is vividly portrayed in Fig. 5C. Concurrently, time-dependent Receiver Operating Characteristic (ROC) curves, based on nomogram-derived risk scores, were delineated. The areas under the curves for 1, 3, and 5 years were quantified at 0.743, 0.724, and 0.708, respectively (Fig. 5D). This confluence of results collectively underscores the robust prognostic predictive capacity embodied in our meticulously constructed model.

In a nuanced exploration of the model’s predictive efficacy, a risk factor plot materialized. This visual representation lucidly elucidates that the low-risk cohort exhibits prolonged survival times and a higher count of survivors. Notably, the heightened expression of all four key LncRNAs in the high-risk group hints at their potential role as risk factors for PRAD (Fig. 5E).

### WGCNA and enrichment analysis of riskscore in prostate cancer progression

**Fig. 6:**
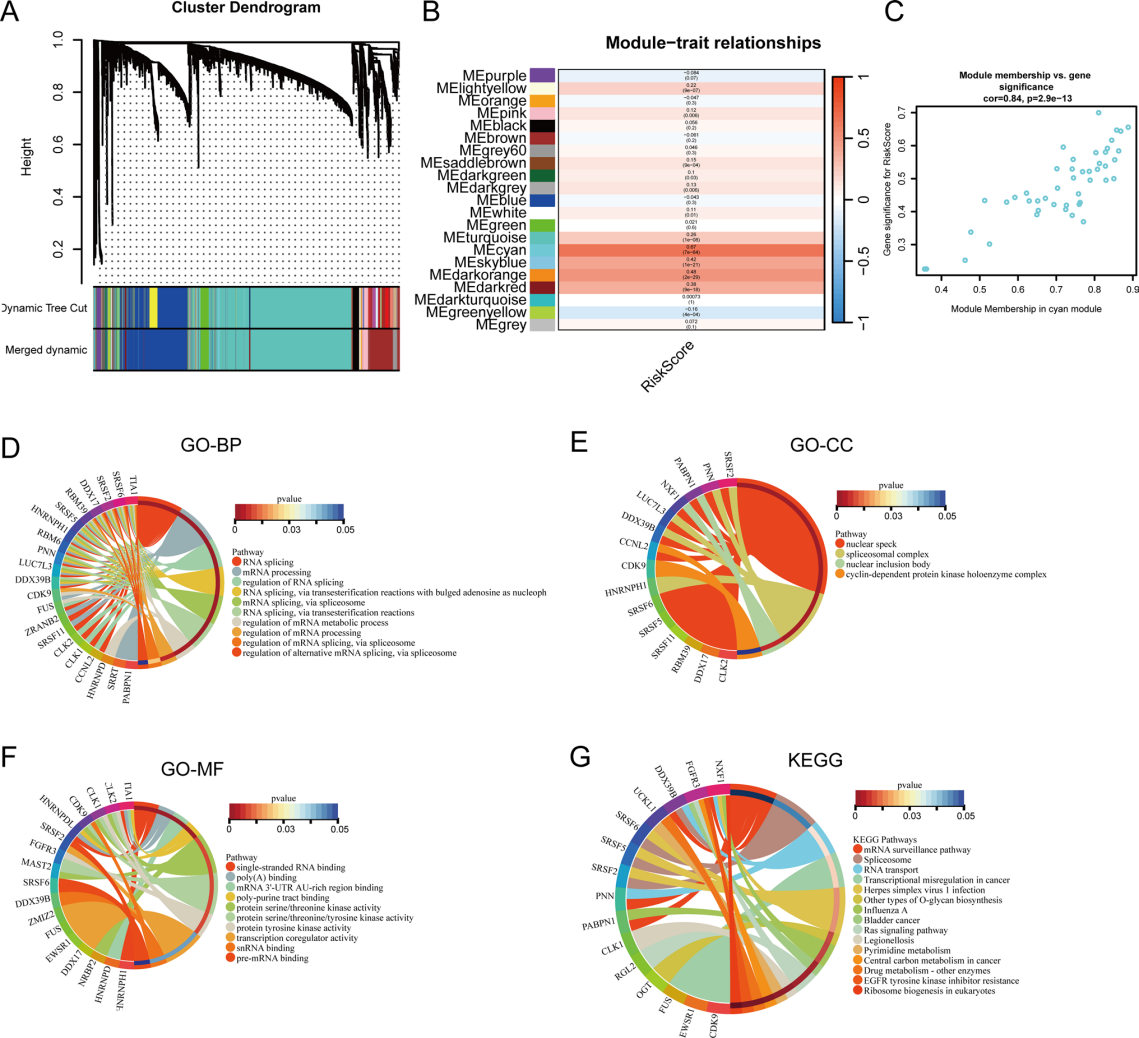
WGCNA and enrichment analysis. **A** Identification of module eigengenes (MEs) associated with riskscore using TCGA-PRAD datasets. **B** Heat map of module-trait relationships. **C** Scatter plot of module membership versus gene significance. **D** GO-BP enrichment analysis for genes in cyan module. **E** GO-CC enrichment analysis for genes in cyan module. **F** GO-MF enrichment analysis for genes in cyan module. **G** KEGG enrichment analysis for genes in cyan module.

To conduct an in-depth exploration of the biological functions intertwined with riskscore and attain a nuanced comprehension of its implications in the progression of prostate cancer, we executed WGCNA on TCGA-PRAD samples, utilizing riskscore as the phenotype data. Employing a soft threshold of 4, the cyan module emerged as the module most profoundly correlated with riskscore (correlation coefficient = 0.67, p = 7e−64) (Fig. 6A, B). The scatter plot within the cyan module accentuates a robust positive correlation between the expression of genes within the module and riskscore (correlation coefficient = 0.84, p = 2.9e−13) (Fig. 6C). This robust correlation underscores the significance of the cyan module genes in the context of riskscore, providing a robust foundation for subsequent exploration.

Subsequent to the identification of the cyan module, we proceeded with an enrichment analysis on its constituent genes to unravel the biological functions associated with riskscore. The outcomes of the enrichment analysis unveiled intriguing associations across various biological aspects. In the realm of Biological Process (BP), riskscore demonstrated substantial associations with RNA splicing, mRNA processing, and the regulation of RNA splicing (Fig. 6D). This suggests a potential regulatory role of riskscore in pivotal processes related to RNA metabolism, indicating its influence on the transcriptional machinery in the progression of prostate cancer. Concerning Cellular Component (CC), riskscore exhibited noteworthy correlations with nuclear speck, spliceosomal complex, and nuclear inclusion bodies (Fig. 6E). Within Molecular Function (MF), riskscore displayed associations with single-stranded RNA binding, poly(A) binding, and mRNA 3’-UTR AU-rich region binding (Fig. 6F). These enrichments shed light on the subcellular localization and functional roles of genes within the cyan module.

In the context of the Kyoto Encyclopedia of Genes and Genomes (KEGG) pathway analysis, riskscore demonstrated significant associations with the mRNA surveillance pathway, Spliceosome, and RNA transport (Fig. 6G). These enrichments furnish insights into potential pathways through which riskscore may exert its influence on the progression of prostate cancer.

### Deciphering the interplay: riskscore and its impact on immune cell infiltration in prostate cancer

**Fig. 7:**
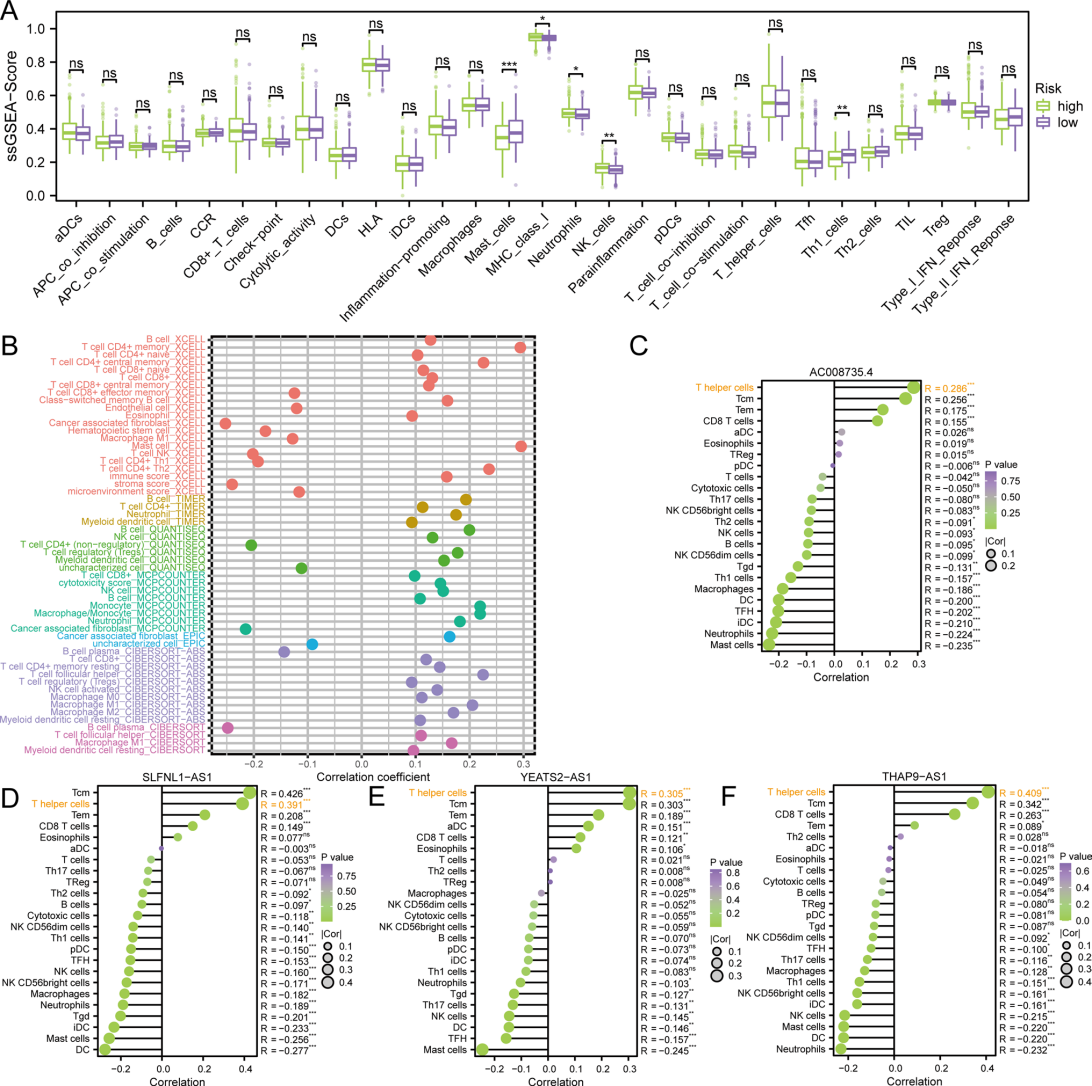
Disclosure of the relationship between riskscore and immune cell infiltration. **A** Box plot of immune cell differences between high and low risk groups. **B** Correlation graph between the predicted immune cell score and riskscore based on eight software. **C** The lollipop graph depicts the correlation between AC008735.4 and immune phenotype. **D** The lollipop graph depicts the correlation between SLFNL1-AS1 and immune phenotype. **E** The lollipop graph depicts the correlation between THAP9-AS1 and immune phenotype. **F** The lollipop graph depicts the correlation between YEATS2-AS1 and immune phenotype

The pivotal role of immune cell infiltration in the tumor microenvironment and its influence on the prognosis of PRAD necessitate an in-depth exploration. To unravel the intricate relationship between our riskscore and immune cell infiltration, we employed the ssGSEA algorithm, utilizing marker genes for 29 immune cell types sourced from the GSEA official website. Scoring each TCGA-PRAD sample, we compared the differences in immune cell infiltration between high and low-risk groups. Remarkably, Mast cells, MHC class I, Neutrophils, NK cells, and Th1 cells exhibited significant differences between these groups, as depicted in Fig. 7A.

Acknowledging the potential bias associated with a single enrichment algorithm, we extended our analysis by employing eight diverse immune infiltration algorithms, including XCELL, TIMER, QUANTISEQ, MCPCOUNTER, EPIC, and CIBERSORT. The results, maintaining a significance level of p < 0.05, demonstrated consistency with the ssGSEA findings, providing robust support for the association between riskscore and Th1 cell infiltration, as illustrated in Fig. 7B.

Given the intricate association between riskscore and various immune cell infiltrates, a more granular analysis was undertaken, focusing on the four lncRNAs implicated in riskscore construction. The outcomes revealed a robust correlation between these lncRNAs and Thelper cells, as depicted in Fig. 7C–F. This observation suggests a pivotal role of the four lncRNAs involved in riskscore construction in influencing the prognosis of PRAD by modulating the presence of Thelper cells within the tumor microenvironment.

### A holistic examination of factors impacting prostate cancer progression

**Fig. 8:**
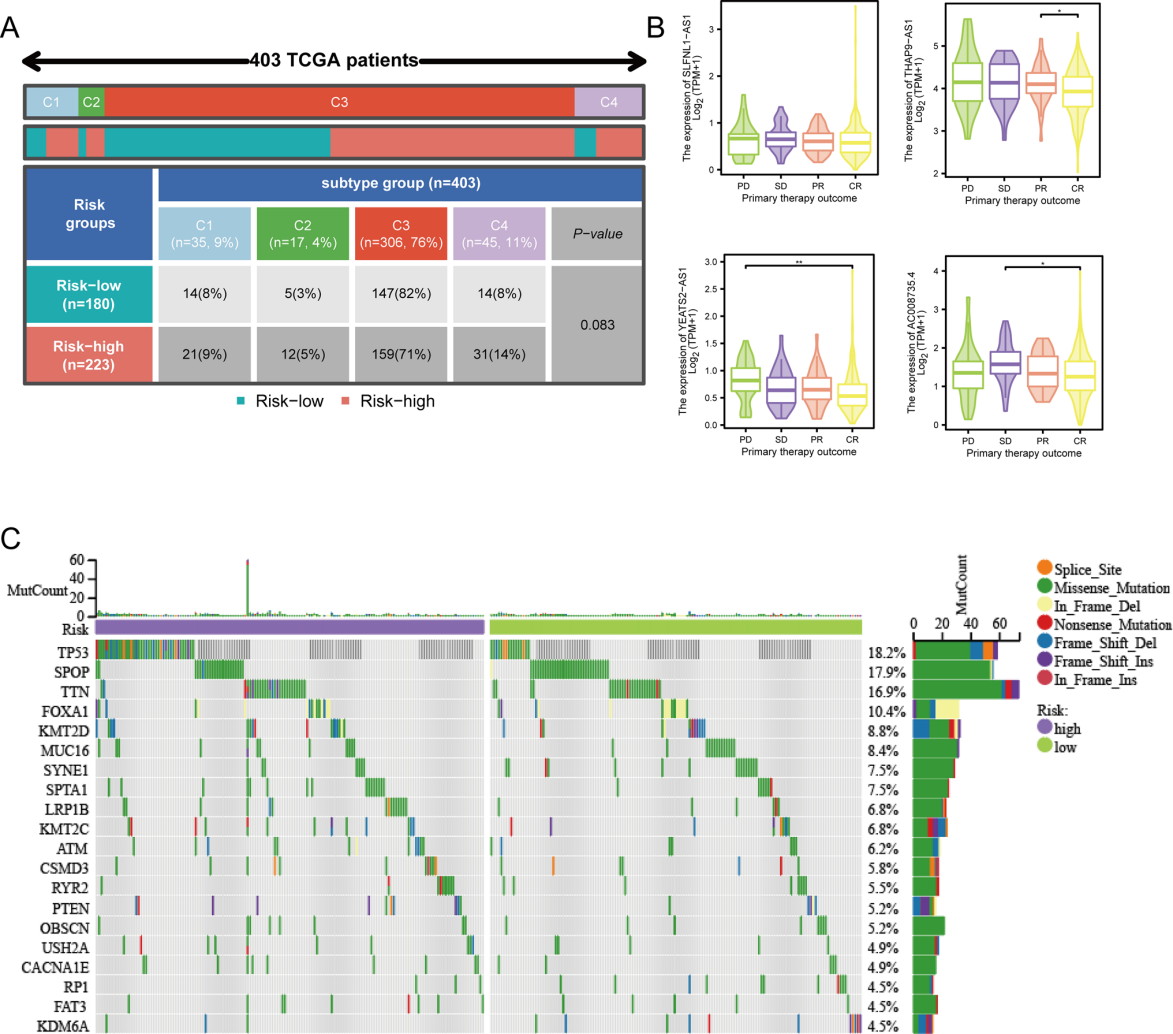
Comprehensive analysis of two risk groups. **A** Immunotyping analysis. **B** Box plot of the relationship between 4 LncRNA and primary therapy outcome. **C** Waterfall plot of mutated genes between high and low risk groups

The majority of patients within the TCGA-PRAD cohort underwent thorough immune response profiling, facilitating a comprehensive analysis of riskscore across immune subtypes. Intriguingly, our investigation revealed no statistically significant differences in riskscore among the C1-C4 immune subtypes. This lack of significance can be ascribed to the inherent heterogeneity observed in the tumor immune microenvironment of prostate cancer patients, as elucidated in Fig. 8A.

To unravel the connection between riskscore and the outcomes of initial treatment in PRAD patients, we scrutinized the expression of the four lncRNAs across subgroups categorized by treatment outcomes—CR, Partial Response (PR), Stable Disease (SD), and Progressive Disease (PD). The findings highlighted notable differences in the expression of three out of the four lncRNAs across distinct treatment outcomes, suggesting potential associations with treatment responses, as depicted in Fig. 8B. This insight accentuates the role of the mitophagy-related lncRNA signature in influencing the diverse responses of PRAD patients to initial treatment.

Recognizing the intrinsic link between gene mutations and cancer initiation and progression, we conducted a comparative analysis of mutational differences between the high and low-risk groups. The waterfall plot vividly illustrated TP53 as the gene with the most pronounced mutations between the two risk groups, as showcased in Fig. 8C. This observation posits a potential connection between the mitophagy-related lncRNA signature and TP53 mutations, implicating its role in shaping the mutational landscape of prostate cancer.

### Significance of YEATS2-AS1 in prostate cancer progression

Building upon the insights garnered from the preceding analysis, we discerned noteworthy distinctions in the expression of YEATS2-AS1 between patients exhibiting a CR and those with Disease Progression (PD). This observation alludes to the potentially pivotal role of YEATS2-AS1 in influencing the prognosis of PRAD. Intrigued by this discovery, we expanded our inquiry to conduct a pan-cancer analysis of YEATS2-AS1, with the objective of unraveling its prognostic impact across various malignancies.

**Fig. 9:**
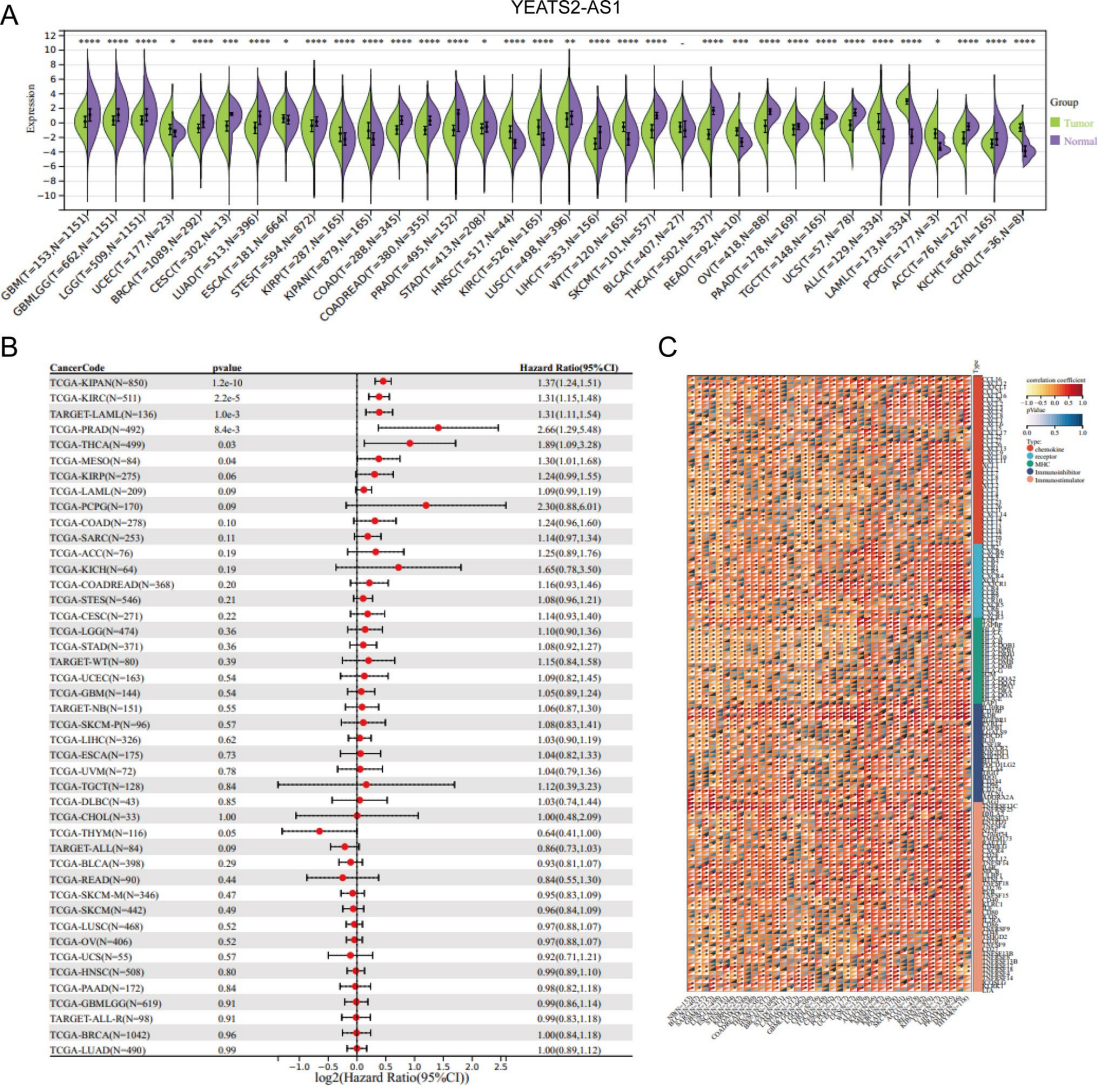
Pan-cancer analysis of YEATS2-AS1. **A** YEATS2-AS1 expression differences in a variety of tumors and normal tissues. **B** Forest plots of YEATS2-AS1 in multiple tumors. **C** Heat map of YEATS2-AS1 correlation with immune-related genes in various tumors

YEATS2-AS1 manifested significantly elevated expression levels in multiple cancer tissues, encompassing PRAD, OV (Ovarian Cancer), and UCEC (Uterine Corpus Endometrial Carcinoma), in comparison to normal tissues, as illustrated in Fig. 9A. This pervasive overexpression pattern implies a potential oncogenic role for YEATS2-AS1 across diverse cancer types. Further exploration into the prognostic relevance of YEATS2-AS1 unveiled its substantial impact on the outcomes of various tumors, including KIRC (Kidney Renal Clear Cell Carcinoma), PRAD, and THCA (Thyroid Carcinoma), as detailed in Fig. 9B. This observation underscores the versatile influence of YEATS2-AS1 in shaping the progression and prognosis of different cancer types.

In pursuit of a comprehensive understanding of the functions associated with YEATS2-AS1 in cancer, we delved into its correlation with immune-related functions across multiple tumors. The analysis revealed associations with chemokine and Major Histocompatibility Complex (MHC), shedding light on the potential involvement of YEATS2-AS1 in modulating immune-related processes, as depicted in Fig. 9C.

### Drug sensitivity based on riskscore in prostate cancer

To offer in-depth clinical insights into the relevance of riskscore in PRAD, we employed the oncopredict package for drug sensitivity analysis on the TCGA-PRAD dataset. The ensuing analysis unveiled noteworthy variations in IC50 values for specific drugs between the high and low-risk groups, providing valuable information for the customization of therapeutic strategies.

**Fig. 10:**
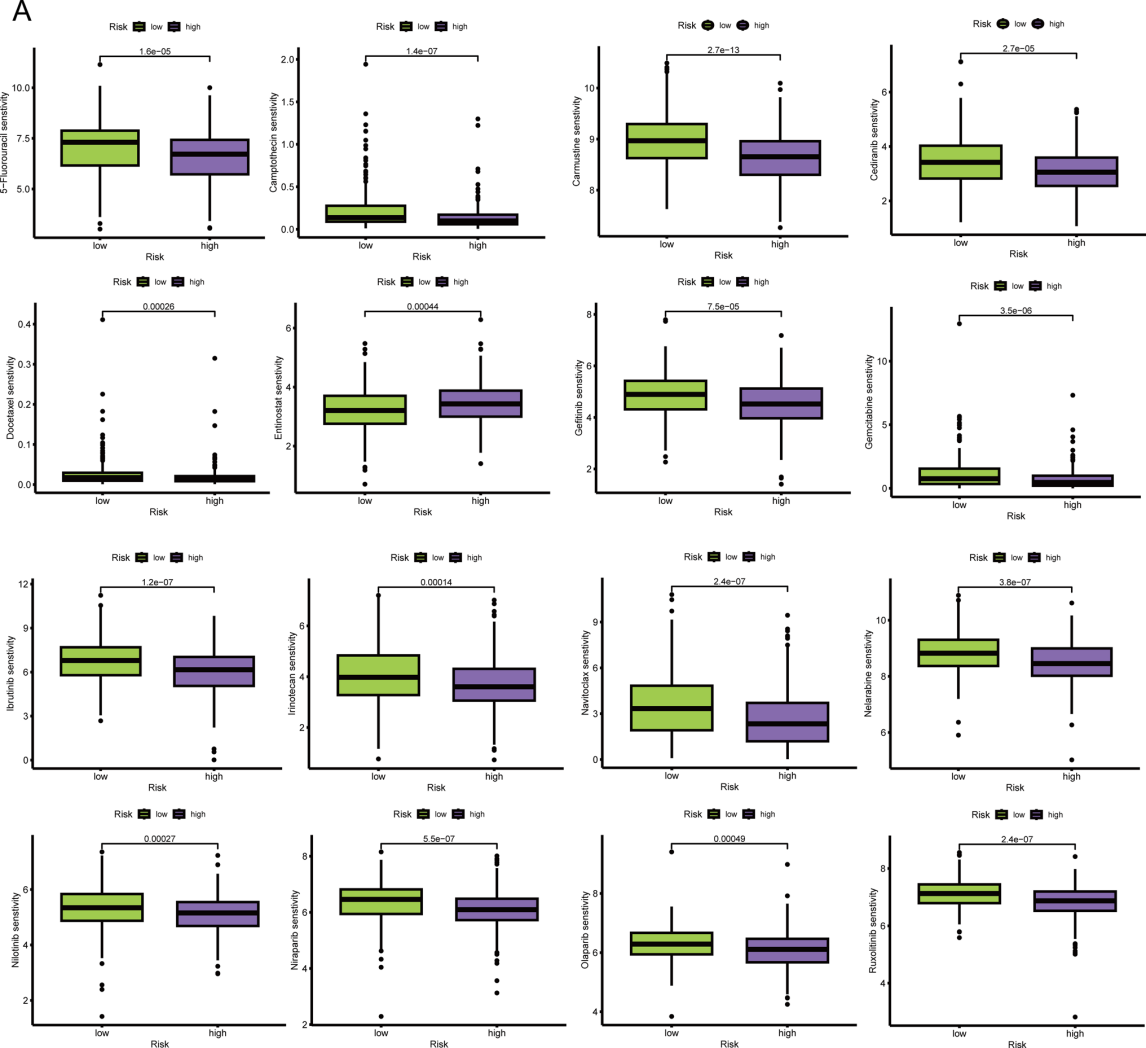
Drug prediction based on riskscore. **A** Boxplot of the difference in drug sensitivity between high-risk and low-risk groups

The analysis discerned drugs with significantly divergent sensitivities between the high and low-risk groups. Particularly, Carmustine and other drugs emerged as potentially more suitable for chemotherapy in high-risk group patients, as evidenced by lower IC50 values. Conversely, drugs like Entinostat demonstrated higher suitability for chemotherapy in low-risk group patients, as depicted in Fig. 10A. Carmustine, identified as more appropriate for high-risk group patients, holds promise in addressing the distinct challenges associated with high-risk prostate cancer. Conversely, the preference for Entinostat in low-risk group patients suggests a tailored chemotherapy approach for individuals with a favorable prognosis.

**Fig. 11:**
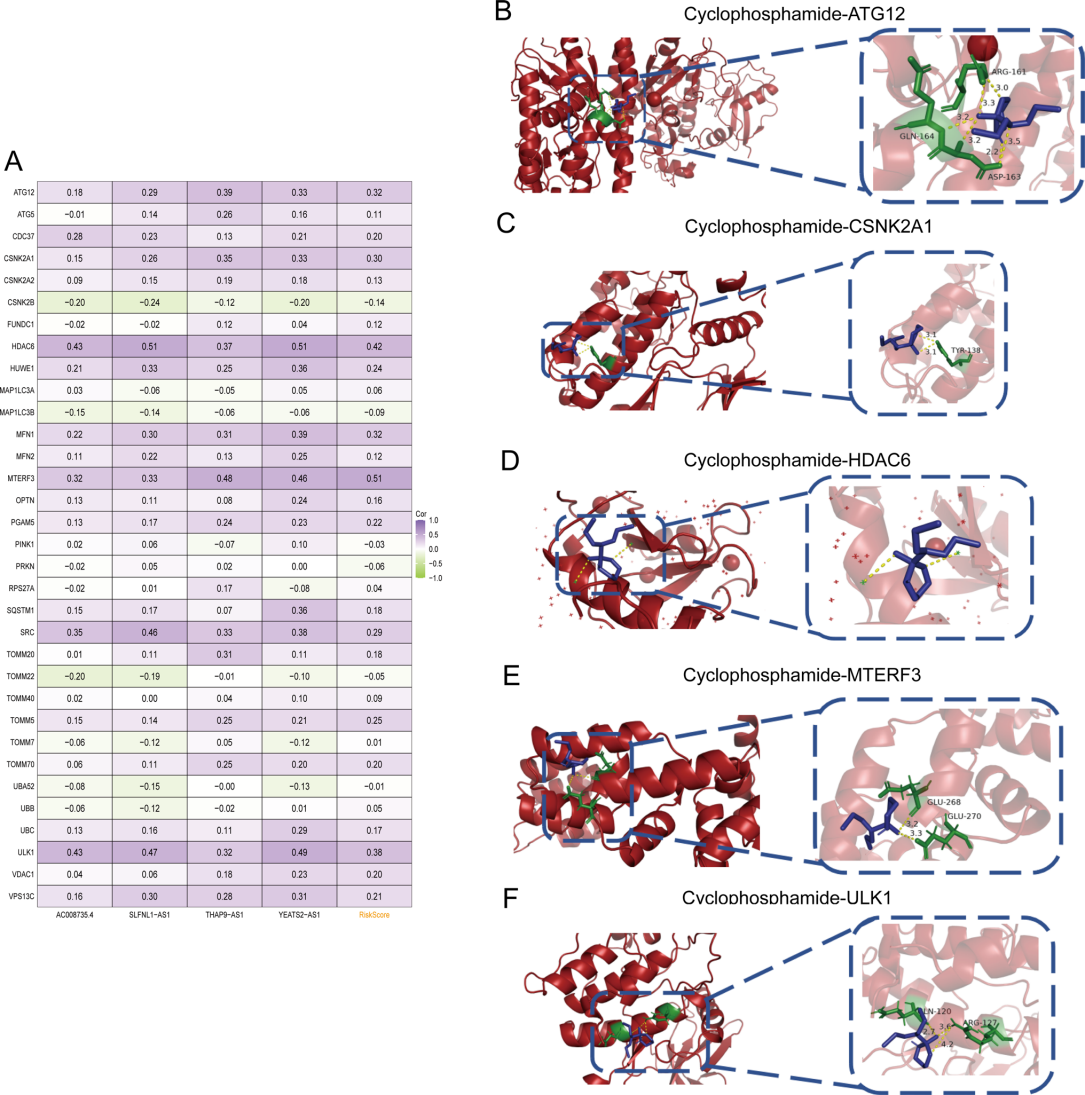
Molecular docking pattern of Cyclophosphamide and mitophagy-related proteins. **A** Heat map of correlation between risk model and mitophagy-related genes. **B** Cyclophosphamide and ATG12. **C** Cyclophosphamide and CSNK2A1. **D** Cyclophosphamide and HDAC6. **E** Cyclophosphamide and MTERF3. **F** Cyclophosphamide and ULK1

Regression Analysis and Molecular Docking of Mitophagy-Related Proteins with drugs to unravel the intricate molecular landscape, we conducted an extensive analysis to elucidate the interrelationships among the identified mitophagy-related lncRNAs, riskscore, and pivotal mitophagy-related genes. Specifically, ATG12, CSNK2A1, HDAC6, MFN1, MTERF3, and ULK1 exhibited a significant correlation with riskscore (correlation coefficient > 0.3), as depicted in Fig. 11A.

Expanding our investigation from the identified drugs in Fig. 10, we directed our focus toward Cyclophosphamide, which demonstrated the highest negative correlation with riskscore. Subsequently, we conducted molecular docking analyses to probe the potential interactions between Cyclophosphamide and the proteins encoded by the aforementioned six genes.

The outcomes of the molecular docking analysis revealed intriguing insights. Five out of the six proteins, namely ATG12, CSNK2A1, HDAC6, MTERF3, and ULK1, demonstrated the capability to form multiple hydrogen bonds with Cyclophosphamide, indicative of favorable binding affinities (Fig. 11B–F). However, MFN1 exhibited distinctive characteristics in its interaction profile.

## Discussion

Prostate cancer, a multifaceted challenge in oncology, necessitates innovative strategies for prognosis, stratification, and treatment [2, 3, 47, 48]. Recently, machine learning has emerged as a promising avenue for decoding the intricate dynamics of cancer, providing a nuanced understanding of molecular intricacies [49–51]. Our study pioneers a machine learning-based integration to develop a mitophagy-related lncRNA signature tailored for predicting the progression of prostate cancer. Mitophagy, a process selectively targeting damaged mitochondria for degradation, plays a pivotal role in cellular homeostasis and has implications in cancer progression. The interplay between mitophagy-related genes and lncRNAs remains unexplored in the context of prostate cancer. Our study bridges this gap, utilizing machine learning to analyze the extensive TCGA-PRAD dataset and unearth a mitophagy-related lncRNA signature with prognostic significance.

Machine learning serves as a powerful tool in deciphering complex biological interactions in our approach, enabling a sophisticated analysis of intricate relationships within vast datasets [52–54]. By leveraging computational prowess, our study aims to unravel the molecular intricacies governing prostate cancer progression. The identification process involved Pearson correlation analysis to discern lncRNAs highly correlated with mitophagy-related gene expression, leading to the identification of 54 PRMR-LncRNAs significantly associated with prostate cancer prognosis. These lncRNAs promise insights into the complex dynamics of prostate cancer progression.

The integration of diverse machine learning algorithms became imperative for refining and validating our mitophagy-related lncRNA signature. The final selection, StepCox[both] + RSF, identified 15 PRMR-LncRNAs with optimized predictive capabilities. This precision-driven approach positions our signature as a robust tool for discerning the intricate landscape of prostate cancer prognosis. The exploration of the functional implications of our 54 PRMR-LncRNAs involved GSVA, providing insights into their association with key biological pathways, notably correlating with the RIG-I like receptor signaling pathway. This functional insight enhances our understanding of the molecular underpinnings and hints at potential therapeutic avenues.

Recognizing the inherent heterogeneity within prostate cancer, NMF clustering led to the identification of four distinct subtypes, each carrying unique prognostic implications [55, 56]. This nuanced subtyping not only presents an opportunity for more personalized prognosis but also hints at diverse molecular landscapes underlying prostate cancer progression. To ensure the clinical applicability of our findings, we constructed and validated a nomogram model based on the expression levels of the four key PRMR-LncRNAs. Calibration curves, Kaplan–Meier survival analyses, and time-dependent ROC curves collectively validated the robust predictive power of our signature, bridging the translational gap from comprehensive molecular insights to actionable clinical tools. However, experimental studies on AC008735.4, SLFNL1-AS1, THAP9-AS1, and YEATS2-AS1 are currently lacking. The roles and mechanisms by which these four lncRNAs contribute to prostate cancer remain unclear. Future research should focus on elucidating the functions of these lncRNAs and uncovering their underlying mechanisms in the pathogenesis and progression of the disease.

WGCNA became pivotal to dissect the interplay between riskscore and various biological functions [57, 58]. Immune cell infiltration analysis illuminated the intricate relationship between riskscore, Th1 cells, and the identified PRMR-LncRNAs, strengthening the reliability of our findings and shedding light on potential immunomodulatory roles. Our exploration extended beyond prognostic insights, delving into the realm of therapeutic tailoring. Predicting drug sensitivities based on riskscore identified drugs suitable for high and low-risk groups. Molecular docking analyses validated potential interactions between Cyclophosphamide and proteins encoded by mitophagy-related genes, suggesting novel therapeutic targets. The integrated findings offer a comprehensive understanding of prostate cancer progression. The machine learning-driven mitophagy-related lncRNA signature, validated clinically, provides not only a prognostic tool but also unveils potential therapeutic avenues. The exploration of immune infiltration and drug sensitivities deepens our understanding of the tumor microenvironment and opens new possibilities for targeted interventions.

Our study not only contributes to the scientific understanding of prostate cancer but also paves the way for clinically relevant tools. The intricate dance between mitophagy-related lncRNAs, immune modulation, and drug sensitivities sets the stage for a new era in precision oncology. Future research endeavors should focus on validating these findings in larger cohorts, exploring the potential for targeted therapies, and further deciphering the functional roles of the identified lncRNAs.

However, there are some limitations to our study. First, our study was largely based on data from TCGA, where most patients were white or Asian. Extending our findings to patients of other ethnicities should be done with great caution. Second, external validation of signature profile and prognosis nomogram in a more independent cohort is warranted.

In essence, our research represents a concerted effort to harness the synergy of machine learning and biological insights, paving the way for a new era in prostate cancer research and clinical management. The integrated approach offers a roadmap for unraveling the complex tapestry of cancer progression and holds promise for advancing personalized medicine in the realm of prostate cancer.

### Supplementary Information

Additional file 1.

## Data Availability

The data could be obtained from the corresponding author.
