# Supplementary figures and images for: Machine learning-based integration develops a mitophagy-related lncRNA signature for predicting the progression of prostate cancer: a bioinformatic analysis
Source: Discov Oncol. 2024 Jul 29;15:316. doi: 10.1007/s12672-024-01189-5 (PMC11286916; doi:10.1007/s12672-024-01189-5)

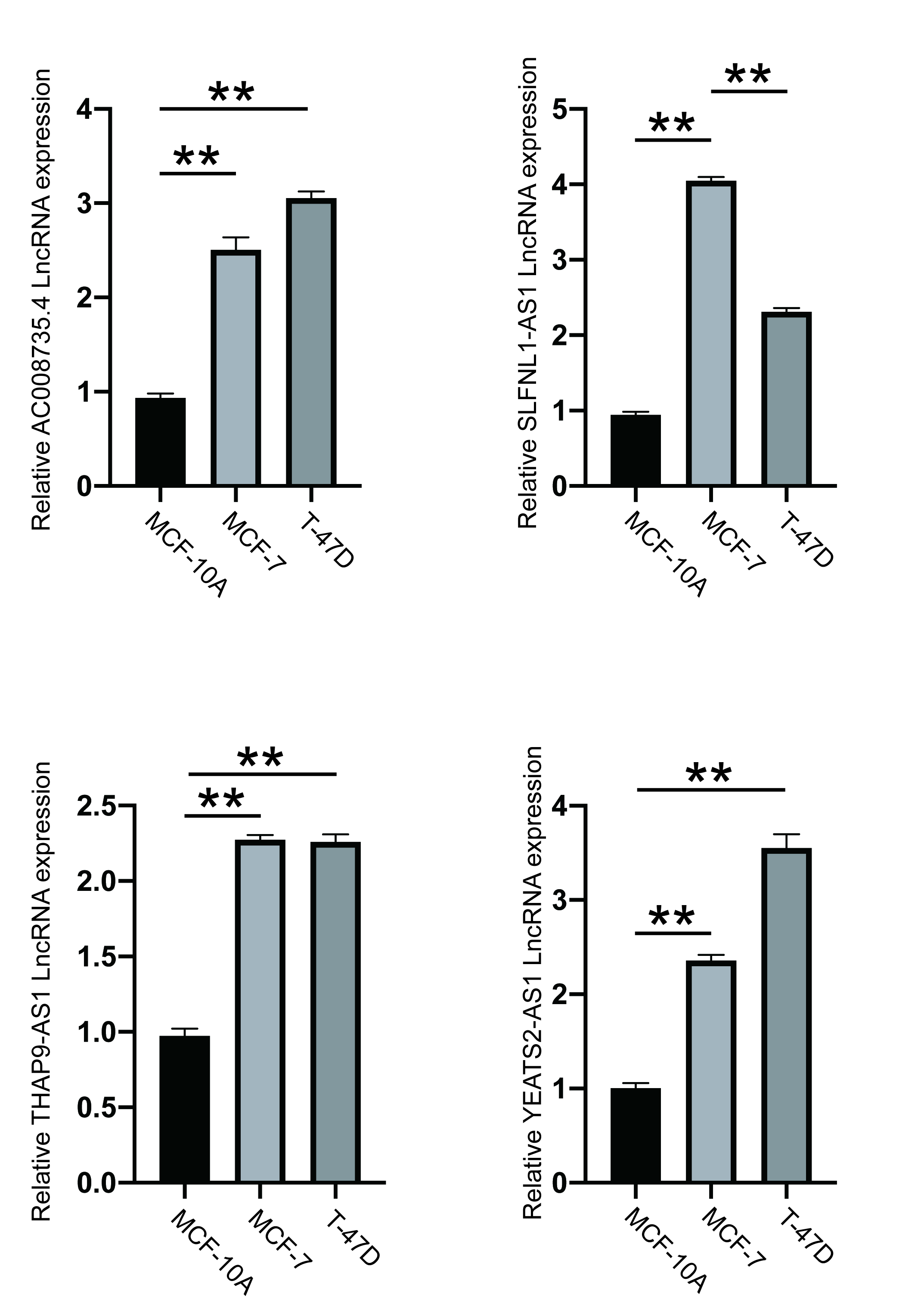

Supplement: Supplementary file 1 — Additional file 1. [file 12672_2024_1189_MOESM1_ESM.tif]
